# Regarding: ‘modified “Xingnao Kaiqiao” acupuncture technique shortens the recovery time of comatose patients’

**DOI:** 10.1080/07853890.2025.2483984

**Published:** 2025-04-03

**Authors:** Yuqi Zhao, Hu Li

**Affiliations:** a Department of Joint and Sports Medicine, Shandong Provincial Third Hospital, Shandong University, 250031, Jinan, China; b Department of Acupuncture-Moxibustion and Tuina, Shandong Provincial Third Hospital, Shandong University, 250031, Jinan, China

Dear Editor

We read with great interest the article by Meng et al. titled ‘Modified “Xingnao Kaiqiao” Acupuncture Technique Shortens the Recovery Time of Comatose Patients’ [1]. Coma, as a severe manifestation of consciousness disorders, imposes significant burdens on patients, families and society. We commend the authors for their valuable contribution to exploring acupuncture as a potential adjunct therapy for coma management. However, we would like to raise several methodological concerns and suggestions to further strengthen the reliability and interpretability of the findings.

## 1. Heterogeneity in patient management across departments

The study enrolled comatose patients from four departments (Neurocritical ICU, Neurosurgery, Cerebrovascular Medicine, and Traditional Chinese Medicine) within a single hospital. In clinical practice, differences in critical care protocols—such as respiratory support, sedative use, and tracheotomy—across departments may significantly influence patient outcomes. For example, patients in the department of traditional Chinese Medicine often undergo aggressive non-pharmacological treatment (e.g. acupuncture), while neurocritical ICU patients may receive more standardized ventilator management. However, Table 1 does not clarify the departmental distribution of patients or whether these protocols were standardized. Such variability could confound the observed effects of acupuncture. We recommend that the authors to disclose the number of patients from each department and compare baseline treatments (e.g. sedatives, tracheotomy rates) across groups, and address whether department specific protocols were adjusted in statistical analyses.

## 2. Ambiguity in key definitions and outcome measures

While the study comprehensively evaluated outcomes (e.g. GCS scores, recovery time), several critical definitions lack clarity. ‘Recovery’ criteria: The threshold for defining recovery (e.g. GCS improvement, discharge readiness) is not explicitly stated. For instance, a GCS increase of ≥2 points is often used to signify meaningful neurological improvement, but this was not specified. ‘Total days of consciousness disturbances’: It remains unclear whether this metric includes prehospitalization or pretreatment days. Clarifying this is essential to avoid misinterpreting the natural history of coma. ‘Treatment duration’: The authors report shorter treatment durations in the acupuncture group (15 vs. 24 days, *p* < 0.001), but factors influencing hospitalization (e.g. insurance policies, family preferences) were not discussed. In China, socioeconomic pressures often shorten hospital stays, which may bias outcomes.

## 3. Inconsistencies in data reporting

Several discrepancies between the text and tables raise concerns:

Post-treatment GCS scores in the section ‘Comparison of general conditions between acupuncture and non-acupuncture groups’: The text states ‘the results revealed no significant statistical differences in gender, age, … post-treatment GCS scores’ (*p* > 0.05), but later claims ‘significant differences in post-treatment GCS scores between the groups’ (*p* < 0.05). This contradiction needs clarification. Cause of coma: The text describes higher incidences of hemorrhage (68.4 vs. 41.4%) and trauma (20.3 vs. 27.6%) in the acupuncture group compared to controls, but Table 1 lists hemorrhage as 41.4 vs. 68.4% and trauma as 27.6 vs. 20.3% in the acupuncture group compared to controls. Clarification is urgently needed. Recovery rates: The text reports 76 patients as recovered, while Tables 1 and 3 in [1] both list 90 patients as recovered totally, which consists of 38 patients in the control group and 52 in the acupuncture group. This inconsistency requires reconciliation. This inconsistency undermines the validity of subgroup analyses.

## 4. Multidisciplinary rehabilitation and standardized protocols

Chronic disorders of consciousness (DoC) require multidisciplinary interventions, including neuromodulation, hyperbaric oxygen therapy, and motor rehabilitation. While acupuncture is weakly recommended in these guidelines, its integration with evidence based therapies (e.g. transcranial direct current stimulation) may enhance efficacy [2,3]. However, the authors did not disclose whether patients received concurrent rehabilitation therapies (e.g. physiotherapy, hyperbaric oxygen), which could confound outcomes. We urge the authors to provide details on adjuvant therapies administered during the study period, and discuss how multidisciplinary collaboration was implemented in their institution.

## 5. Conclusion

This study provides preliminary evidence supporting acupuncture as a time-shortening intervention for coma recovery. However, clarifying departmental protocols, standardizing outcome definitions, resolving data inconsistencies, and disclosing multidisciplinary treatment details are crucial to validate these findings. We hope the authors address these concerns in subsequent work, paving the way for high quality RCTs that integrate acupuncture into holistic comatose management protocols.

## Data Availability

Not applicable. No new data were created or analysed for this article.
